# Reproducible image handling and analysis

**DOI:** 10.15252/embj.2020105889

**Published:** 2021-01-22

**Authors:** Kota Miura, Simon F Nørrelykke

**Affiliations:** ^1^ The Network of European Bioimage Analysts (NEUBIAS); ^2^ Nikon Imaging Center University of Heidelberg Heidelberg Germany; ^3^ ScopeM ETH Zurich Zurich Switzerland

**Keywords:** Methods & Resources

## Abstract

Image data are universal in life sciences research. Their proper handling is not. A significant proportion of image data in research papers show signs of mishandling that undermine their interpretation. We propose that a precise description of the image processing and analysis applied is required to address this problem. A new norm for reporting reproducible image analyses will diminish mishandling, as it will alert co‐authors, referees, and journals to aberrant image data processing or, if published nonetheless, it will document it to the reader. To promote this norm, we discuss the effectiveness of this approach and give some step‐by‐step instructions for publishing reproducible image data processing and analysis workflows.

## Graphical abstract

Leading experts describe how systematic description of processing and analysis applied to microscopy data is crucial for the proper interpretation of published image data and helps support reproducibility and data integrity.

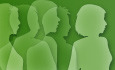

Mishandling and misconduct based on image data in published articles have been surfacing across the life sciences community. The Journal of Cell Biology, reported that 10% of accepted papers contained inappropriate manipulation on image data (Martin & Blatt, 2013). EMBO Press has instituted a three‐tiered classification for image aberrations to handle problems in image data submitted to their journals (Pulverer, 2015). Under this classification, consistently about 20% of post‐review papers presented some sort of problem that required follow‐up by the editorial office. This includes unintentional mistakes in handling images, not affecting the results presented. A manual survey of 20,000 biomedical papers found that 4% had inappropriately duplicated images and 1.9% had deliberate manipulations (Bik *et al*, 2016). Among 99 biomedical papers that were labeled with the “Editorial expression of concern”, 40% had issues with image data (Vaught *et al*, 2017). 760,000 papers sampled from PubMed Open Access Subset were automatically screened and then manually annotated for image data reuse. Of all papers, 0.6% were scored as falsely reusing images (preprint: Acuna *et al*, 2018).

To prevent these problems, in our view, the most effective way is to advocate and practice the scientific norm of reporting methods in a way that renders the experiments reproducible. We first review some of the most common problems seen in image data and analysis in the biosciences. We then argue that the most effective way to prevent these aberrations is by documenting and reporting reproducible image handling and analysis. We provide a step‐by‐step protocol for publishing reproducible image data analysis and figures. Finally, we recommend what journal editors can do to facilitate the submission of reproducible methods. This includes author self‐evaluation of the level of reproducibility of the image analysis methods. Some of the terms we use are subject to variations and ambiguity in their usage; Box 1 defines our use of these terms, “Mishandling”, “Misconducts”, and “Reproducibility”.

As image analysts at two major imaging facilities, we are regularly asked to replicate the typically vague methods in published papers and find this task ranges from straight‐forward, over pleasantly challenging, to impossible.

**Box 1. Definitions of Terms We Use**

**Definitions of Terms**

**“Mishandling” and “Misconduct”**

Throughout the text, we use the word “mishandling” to describe the wrong handling of image data in general, including analysis and figure creation. “Misconduct” describes researchers publishing scientifically wrong results, regardless of the cause being *accidental* or *intentional*. "Accidental" is like driving your car (your scientific project), unaware that you have no thread left on your tires—you are a potential danger to yourself, your car, and the rest of traffic.

"Intentional" should be compared to knowingly installing defeat‐devices in your car to, illegally, pass the exhaust‐regulations in your state. Mishandling of image data might be happening locally and personally. Misconduct is a public affair: Publication of mishandled image data is misconduct.

**“Reproducibility”**

Reproducibility, repeatability, and replicability are terms often used interchangeably. To fix our nomenclature, we will follow Goodman *et al* (2016), who distinguished three types of reproducibility in terms of experiments and data analyses:

1. *Methods* reproducibility: The original meaning of reproducibility (Claerbout & Karrenbach, 1992). The ability to obtain the exact *same* results, by implementing procedures using the *same* data and tools.
2. *Results* reproducibility. Also known as replication. The ability to produce *similar* results, in an independent study, following *similar* procedures and using *similar* tools.
3. *Inferential* reproducibility. The ability to reach the same scientific conclusions by conducting an independent study, or re‐analysis of the data, potentially using *different* tools and methods.

Because the focus of this commentary is on analysis and methods, we are concerned mainly with *methods reproducibility*. This is also the only type of reproducibility that can, feasibly, be verified before publication. We are of the same opinion as Mendes, 2018 that “Scientific journals should not publish non‐reproducible research and thus should promote, or even enforce, such actions”—the actions we have in mind are discussed in this commentary.

Results reproducibility, or rather the lack thereof, has received much attention lately (Baker, 2016). With a reported 50% of preclinical studies not being replicable the economic cost, attributable to irreproducible data analysis and reporting alone, is estimated at $7B/year in the US (Freedman, Cockburn, & Simcoe, 2015). One of four broad strategies to improve reproducibility, as recommended in (Freedman, Venugopalan, & Wisman (2017), is open access to data and methods.

The most infamous recent example, of lacking results and inferential reproducibility, is, arguably, reporting a ~90% rate of irreproducibility of landmark preclinical studies. Only six out of 53 could be reproduced (Begley & Ellis, 2012).

## Image Manipulation—Cause and Consequence

### Mishandling: accidental or intentional

Two major causes underlie image mishandling the first type is caused by a lack of knowledge about the appropriate scientific handling of image data. When processed based on subjective impressions, image data often lose quantitative information (“photoshopping” or “beautification”). Such processing may lead to the misinterpretation as researchers instinctively tend to accentuate the findings. Moreover, without some knowledge of image processing and analysis algorithms, the analysis workflow cannot become reliable enough to establish scientifically valid results, as is the case for any other instrument used in scientific research. Images or analysis results based on wrongly processed images may become included in publications and mislead the research community. We refer to these types as the "accidental” type of mishandling.

The second type is due to *deliberate* manipulations by researchers with the clear goal of supporting their conjectures, at the cost of skewing the data. We refer to these acts as the "intentional” type of mishandling.

### Inappropriate figure creation and image analysis

**Figure 1:**
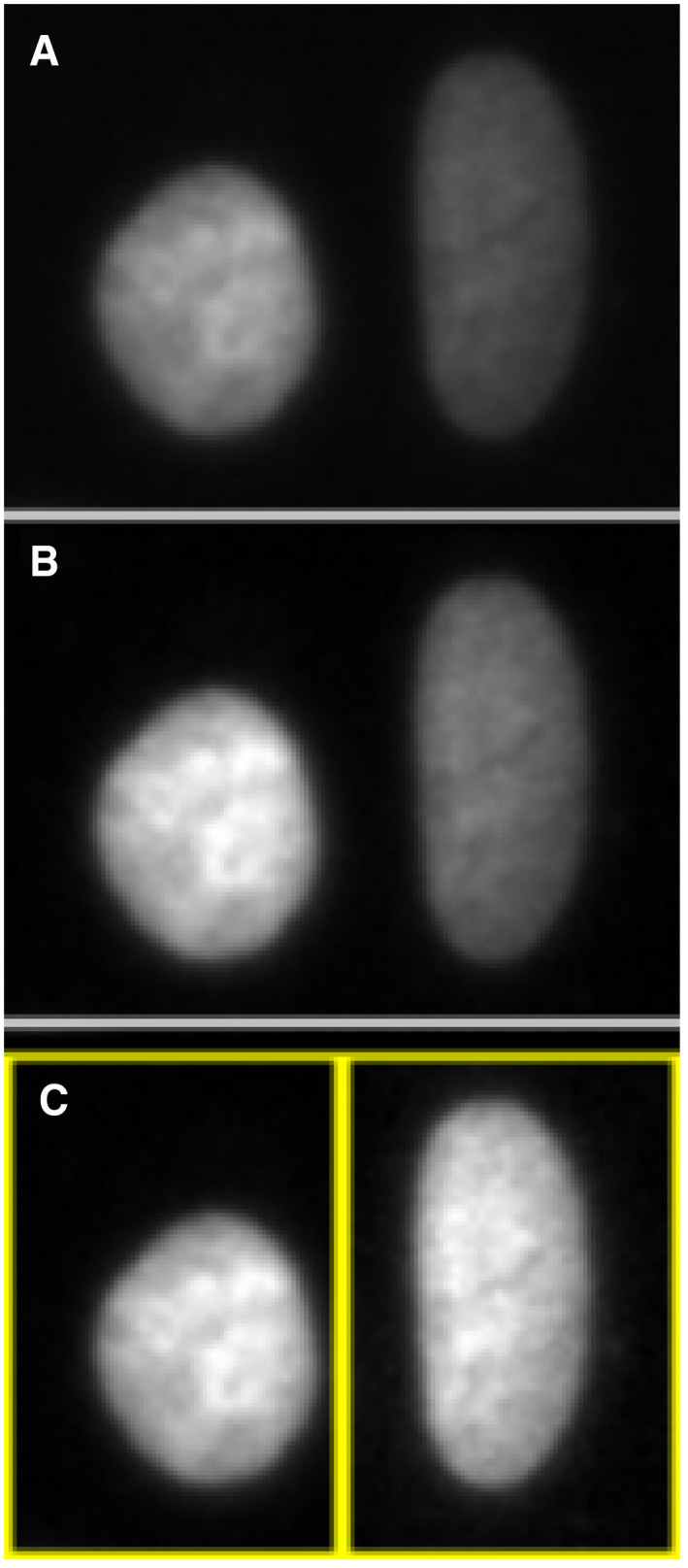
Enhancing contrasts in a wrong way Two Hoechst‐stained nuclei were cropped from the original sample image (A). The full image was contrast‐enhanced using the ImageJ “Enhance Contrast” function (B). The default saturation value was used (0.35%). Both nuclei are now with a better contrast against the background, while preserving the difference in the signal intensity observed in the original image. For comparison, each cell was individually selected and contrast‐enhanced separately indicated by yellow region‐of‐interests (C). The applied function and parameters were exactly the same as B, but the two nuclei now appear to have similar signal intensity. Since the degree of enhancement varies depending on the image, such difference in the processing results happens, depending on the area to which one applies the function. An example of such a case can be found in Fig S12 of Tanno *et al* (2019), available from the Broad Bioimage Benchmark Collection (Ljosa *et al*, 2012; “BBBC039: Nuclei of U2OS Cells in a Chemical Screen” n.d.). No scale bar information was available. The reproducible workflow for the figure shown above is available at: https://github.com/miura/reproducible_bioimage_analysis.

Mishandling often involves changes to the visual rendering of image data that trick the eyes of reviewers and readers. These manipulations can involve duplication of part of an image or a whole image (“cloning”), removal of undesired signals (deletion and biased cropping), insertion of artificial objects, pasting objects to appear near each other or localized changes of image contrast (Rossner & Yamada, 2004). Regardless of the motivation, we refer to mishandling by changing the visual impression as “*figure mishandling*”. For example, enhancing the contrast of image data is generally acceptable if (i) it is applied for segmentation or to create the figures, (ii) no intensity quantification is involved after the operation, (iii) it is applied equally to all conditions, and (iv) it is clearly stated in the manuscript (Johnson, 2012). Condition no. 3 is sometimes ignored, either due to “accidental” or “intentional” actions of the authors. In Fig 1, we demonstrate how one can end up in uneven contrast enhancement depending on how one processes image data (Fig 1A, original image; adjusting contrast Fig 1B, altogether; Fig 1C, individually). Design and layout suggestions for clear and effective figures can be found elsewhere (Schmied & Jambor, 2020; preprint: Jambor *et al*, 2020) and references therein.

On the other hand, mishandling of image data during analysis—not just during the creation of a figure, is another prevalent problem in life science research that is rarely discussed. We illustrate mishandling by wrongly designed image analysis workflows with three representative examples below. We call this “*analysis mishandling”*.

### Analysis mishandling: overuse of single channel image

In some cases, a single fluorescence channel is used both for the object segmentation and for the measurement of the intensity of that object. Without careful workflow design, such an overuse of single channel data yields wrong results. In the worst case, cells with a weak signal are not detected, and hence not measured, unintentionally removing them from the analysis.

**Figure 2:**
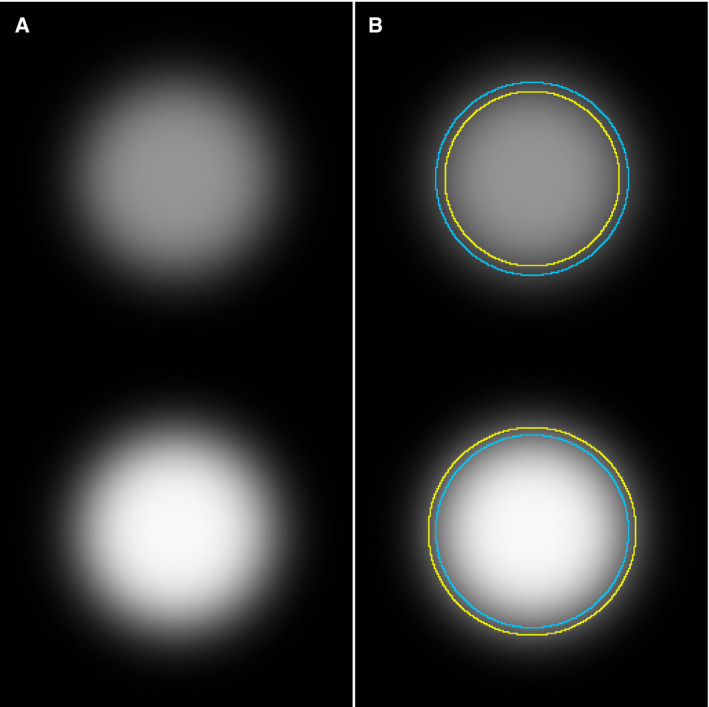
Overuse of single channel image (A) Two circular spots were plotted in an 8‐bit image. The spots are with the same radius, but with different gray values: One is 150 (top, darker) and the other is 250 (bottom, brighter), and then Gaussian blurring was applied to mimic realistic image data. (B) The image, with *both* objects in it, was segmented by automatic global thresholding (Otsu, 1979) to detect the boundary of each and shown as yellow ROIs. The threshold value determined by Otsu’s algorithm was 86 (same threshold applied to both the darker and the brighter circle disk). The mean intensities measured inside the yellow circles were 125.0 and 180.6 for dark and bright circles, respectively. The original boundaries, before applying the Gaussian blur filter, are shown as blue circular ROIs. The workflow for this image analysis, including the creation of the figure above, is available at: https://github.com/miura/reproducible_bioimage_analysis.

In Fig 2, we illustrate a more subtle effect using synthetic image data. Consider two circular spots with different intensities as shown in Fig 2A. The task is to measure the mean intensity of each of these spots. A typical workflow for this task is to segment these spots using intensity thresholding, to delineate each object as region‐of‐interest (ROI), and then to measure the intensity in each ROI. The resulting ROIs are shown as yellow circles, and the original boundary of each spot is shown as blue circles Fig 2B. A larger area was detected with the brighter spot because it has more pixels with intensity values larger than the threshold value. Similarly, the darker spot had a smaller area detected than the input. This difference in the size of the area affects the apparent decrease and increase of the mean intensities compared to the expected values. As the thresholded region is determined based on the signal intensity, the intensity measured in that region is affected by the intensity itself—*the measured intensity depends on the region which depends on the intensity*. Such logical bootstrapping can cause under‐ or over‐estimation of measured values. It is thus preferred that another probe is used and captured in a second channel specifically for segmentation of objects.

### Analysis mishandling: bit depth conversion and normalization

**Figure 3:**
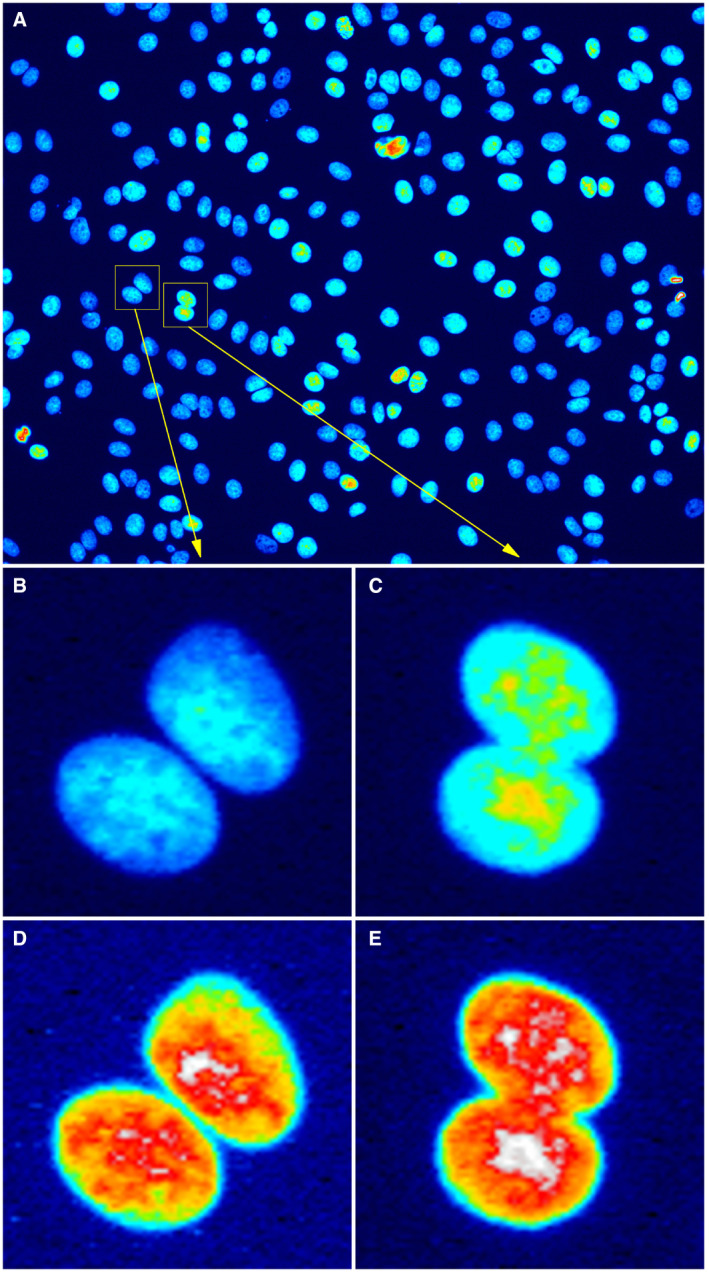
Bit depth conversion and normalization Panel A shows an 8‐bit version of a 16‐bit image, created by conversion in ImageJ/Fiji (Schindelin *et al*, 2012) with the default settings of the software. The display range is automatically set, when opened, based on the pixel intensity distribution of that image, and used when performing the linearly scaled conversion from 16 to 8 bit, of all values inside the display range. Panels B and C are likewise 8‐bit versions, created by cropping the original 16‐bit image, using its display range when converting. Panels D and E are again 8‐bit images, created by letting ImageJ/Fiji automatically determine their individual display ranges, and then making the conversion; thus mimicking the procedure used if two original images are opened and converted independent of each other. This result is similar, but not generally identical, to what we would find if we had applied auto‐contrast or histogram normalization on the two cropped images independent of each other (Fig 1). The “royal” lookup table (LUT) was used, to better visualize the difference. The image is from the publicly available image set BBBC021v1 (Caie *et al*, 2010), available from the Broad Bioimage Benchmark Collection (Ljosa *et al*, 2012). No scale bar information was available. Macro for cropping and generating panels for this figure is available in the GitHub repository at: https://github.com/miura/reproducible_bioimage_analysis. Composite figure was created using the ImageJ/Fiji plugin ScientiFig (Aigouy & Mirouse, 2013).

When a 16‐bit image is converted to an 8‐bit image, the normalization of pixel intensity values depends on the preference setting of the software used. Such a bit depth conversion is likely to be normalized at a different degree depending on the content of the image, i.e., depending on whether it is a dark or a bright image, so the intensities of converted images can no longer be directly compared (Fig 3). Two nuclei, one (Fig 3B) less bright than the other (Fig 3C), are cropped from a single image (Fig 3A) of DAPI stained cells. If these cropped images are handled independent of each other when converted to 8‐bit representation, they may appear to have similar brightness (Fig 3D and E), whereas in reality they have significantly different brightness. Some researchers do not recognize this artifact and present the comparison as a quantitative difference, which is invalid.

### Analysis mishandling: “PSF volume”

**Figure 4:**
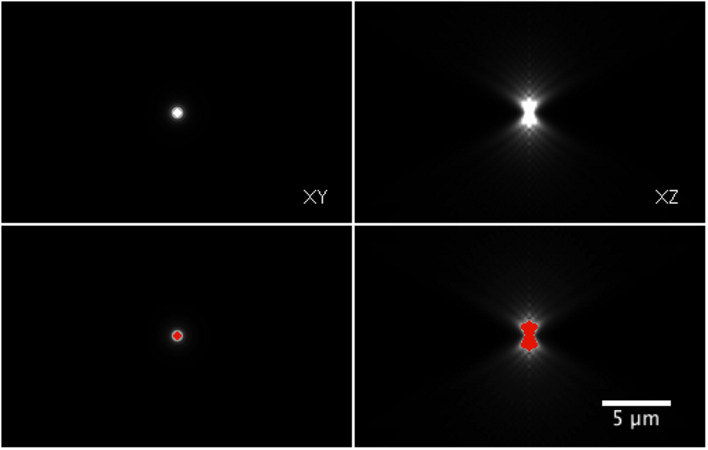
“PSF volume” A 3D image of PSF was made using a PSF generator plugin for ImageJ (Sage *et al*, 2017), showing the Rochard & Wolf model with default parameters provided in the plugin. A point source fluorescence signal with its size below the pixel resolution (100 nm) can be measured with its apparent “volume”. XY (top‐left) and XZ plane (top‐right) at the position of the point source showing the PSF. Image thresholding segments a region that can be measured as “volume” (bottom panels). Details about the generation of this PSF and the reproducible workflow for the composite image shown are available at: https://github.com/miura/reproducible_bioimage_analysis.

Even when the real size of the point source, here a fluorescent signal, is below the resolution limit of the optical setup (diffraction limited spot), the emitted signal propagates in space according to the so‐called impulse response of the imaging system and gives rise to the appearance of the Point Spread Function (PSF). As this results in voxels with non‐zero intensities, some researchers take this as truly occupied space and try to measure this volume as the size of the target signal. It is technically possible to perform this measurement, but it is scientifically wrong (Fig 4).

### The cause of mishandling

It seems that figure mishandling and analysis mishandling are both caused, firstly, by the frequent lack of image analysis training in the life sciences, and, secondly, by the absence of standards for image handling and reporting. Without training and standards, neither the researchers nor the reviewers are able to assess the scientific validity of the image figure and analysis workflow. As a result, many published papers include images with insufficient documentation of the underlying data processing. The mishandling, once published, can become a misconduct. Mishandling *during* image data acquisition is another issue (Marqués *et al*, 2020); here, we focus on problems in data handling *after* the acquisition.

The poor documentation of image handling and analysis may be deep‐rooted: Images have strong and immediate cognitive impact and compel us to accept them as self‐explanatory, even in the absence of a scientific description of methodological details: When modern biology began in the 18th century, with taxonomy, scientific discoveries were often documented using hand‐drawn illustrations. This classic tradition, of images serving merely as illustrations of scientific observations, seems to survive to this day. At the same time, these characteristics are a hindrance to the handling of images as numerical data (Cromey, 2013), and amount to “analysis mishandling”. In our experience, even just a few days of training in the quantitative handling of image data and analysis strongly mitigates this type of scientific misreporting. Integration of bioimage analysis in the curriculum of life science departments, as early as the undergraduate level, could be a powerful solution.

## How Can We Prevent Misconduct?

Guidelines for image data handling and processing may avoid some mishandling and misconduct. While setting rules that apply universally is a difficult task, there have been attempts to propose guidelines for image data acquisition, processing, and analysis (Cromey, 2013). These guidelines capture some of the current best practices in the field. In a similar manner, some journals provide author guidelines suggesting “Do’s and Don’ts” in submitted image data^1^. Such guidelines are effective in setting a baseline for minimal standards and can be effective in avoiding some of the “accidental” type of misconduct (Abbott, 2019).

The limitation of a list of “Do’s and Don’ts” is the unavoidable overgeneralization. Indeed, imaging technology is advancing at a rapid pace, both in terms of hardware (e.g., super‐resolution localization microscopy) and data analysis techniques (e.g., deep learning based approaches), and guidelines are destined to be lagging behind technological innovation. Eventually, an unwieldy, ever‐growing rule book would be required to cover image data handling and analyses comprehensively; it is doubtful this rule book would be used by those most likely to mishandle scientific images. Moreover, as illustrated here, misconduct can go much beyond simple figure falsification and extends to image analysis that is not necessarily apparent in image figures.

We also see that a side effect of “Do’s and Don’ts” lists is that researchers with minimal knowledge in image analysis tend to over‐interpret them. For example, we witnessed many cases where researchers avoided enhancing the contrast of image data altogether, undermining the analytical approach to the image data (see Fig 1). Simplified lists of rules can therefore backfire if they are misused or hinder the dissemination of scientific research.

### Reporting transparency

Instead of setting overly prescriptive rules for image data handling, we propose to report fully reproducible image analysis workflows with all the processing steps used to go from the initial image to its quantification or to figures. By doing so, the results presented in a paper can be reproduced by peers and reviewers. When the reproducibility is secured, accidental mistakes can be more easily uncovered during the reviewing process or by the readers of the paper. The obligation to publish reproducible workflows may also induce a stronger motivation of researchers to learn quantitative image analysis, resulting in a reduction in accidental image manipulation.

**Figure 5:**
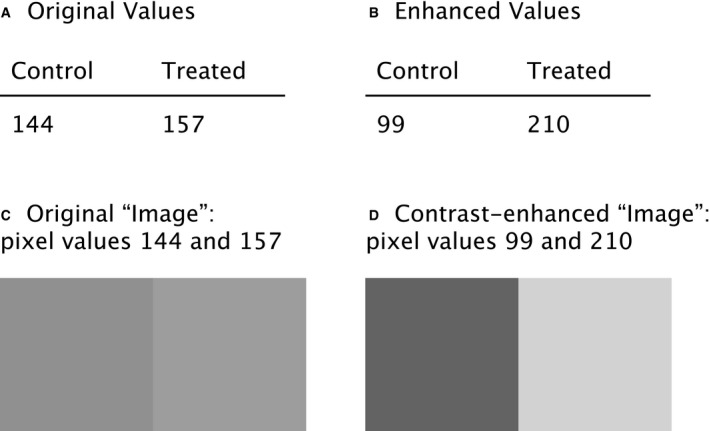
Manipulation of images is like replacing table values (A) Original values of measurement. (B) Manipulated values of values of (A) showing two‐fold increase in treated samples compared to control samples. This is equivalent to enhancing the contrast of images (C) to (D). Note that we are not arguing against the use of contrast enhancements of image data, e.g., histogram stretching, in research papers, merely warning against its inappropriate application. See Fig 1 for more details.

For intentional misconduct, rules are only effective as an additional hurdle that may be weighed against the expected rewards of a high impact publication (Cyranoski, 2006). Intentional misconduct happens across disciplines and data types and irrespective of rules. The abuse of data has a longer history than the recent advancement of the imaging techniques. We know that there have been cases of deliberate manipulations of values listed in tables, reaching probably as far back as Gregor Mendel (Fisher, 1936). Images can in fact be understood as a type of table. We show this with the following example. Let us say that there is a table presenting two different measurements values, one for the control and the other for the treated condition (Fig 5A). Those numbers can easily be adjusted to enhance the difference for better support of one’s hypothesis (Fig 5B). As a two‐cell table can also be thought of as a two‐pixel image (Fig 5C), this artificially enhanced difference is comparable to enhancing the contrast of an image (Fig 5D). Digital image data are just another way of reporting measurement values beside classic tables. Many of the intentional modifications of image data in fact represent a form of data manipulation.

We will be better off by asking researchers to document all the steps they took, to allow others to test the results by reproducing the analysis. The norm of publishing reproducible workflows will hinder the intentional misconduct as it becomes more difficult to hide any manipulation step when the workflow can be critically reviewed and examined. In the following section, we explain practical steps that can be taken to publish reproducible image analysis workflow. These recommendations are what we currently think are the best in terms of openness, popularity, and accessibilities, but we welcome criticism, suggestions, and advice through discussions in the online forum^2^.

## How to Publish a Reproducible Bioimage Analysis Workflow

Reproducible analysis is critically important in data‐driven quantitative sciences (Baker, 2016). We provide below separate recommendations for researchers and for journal editors to prepare a reproducible bioimage analysis workflow.

## Recommendations for Researchers

We define a reproducible image analysis workflow as a set of resources that allow any person to replicate the process of image handling and analysis. The aim is to derive the same results as the authors presented. It has three essential elements:

● the workflow code
● the workflow description
● the original image data.

We call this set the “workflow package”. In the following sections, we explain how each of these elements can be prepared, even without knowledge of computer programming, and how the workflow package can be published.

### Workflow code: documenting your workflow

Before beginning the processing and analyzing of image data, we strongly recommend to examine if the software package to be used supports the generation and export of reproducible analysis workflows (see Box 2: A three‐tiered ranking of software’s support of reproducibility). Some packages provide only a graphical‐user interface (GUI), without allowing the user to export the workflow as a reusable and shareable file. Though reproducible in theory, in practice such GUI‐only software is not ideal for the creation of reproducible workflows as the user would need to manually note each step of the workflow; for example, the name of the menu item selected, which option from the menu was selected and in which order, the exact parameter values that were used, the size, and coordinates of the region‐of‐interest.

**Box 2. A three‐tiered ranking of software’s support of reproducibility**

There are three levels of reproducibility that a software may support. A brief description of what we consider essential requirements for each level as well as a few illustrative examples are given (this is not an exhaustive list).

- Highest degree of reproducibility supported
  - Description (fulfills all or most): Widespread; free; GUI workflows recordable; full scripting capability in one or more common programming languages
  - Examples: Fiji/ImageJ (Schindelin *et al*, 2012); ICY (de Chaumont *et al*, 2012); CellProfiler (McQuin *et al*, 2018); ilastik (Berg *et al*, 2019); QuPath (Bankhead *et al*, 2017); Python; R (Ripley, 2001); fully documented and self‐contained code in a public repository
- Somewhat supportive of reproducibility
  - Description: Locally common; commercial (requires paid license for full access); scriptable, but in proprietary or local language;
  - Examples: Imaris; Amira; Arivis; MATLAB; MetaMorph; Zeiss ZEN; undocumented code with rare dependencies, available in repository
- Not or almost not supportive of reproducibility
  - Description: Rare; commercial; not scriptable (GUI only)
  - Examples: Photoshop, PowerPoint, undocumented code living only on one computer

**URL Links**

**Table:** 

Software	URL
Amira	http://thermofisher.com/amira‐avizo
Arivis	https://www.arivis.com/en/imaging‐science/imaging‐science
CellProfiler	https://cellprofiler.org
ICY	http://icy.bioimageanalysis.org
ilastik	https://ilastik.org
ImageJ/Fiji	https://imagej.net
Imaris	https://imaris.oxinst.com
QuPath	https://qupath.github.io
MATLAB	https://www.mathworks.com/products/matlab.html
MetaMorph	https://www.moleculardevices.com/products/cellular‐imaging‐systems/acquisition‐and‐analysis‐software/metamorph‐microscopy
Photoshop	https://www.adobe.com/products/photoshop.html
PowerPoint	https://office.live.com/start/powerpoint.aspx
Python	https://www.python.org
R	https://www.r‐project.org
Zeiss ZEN	https://www.zeiss.com/microscopy/int/products/microscope‐software/zen/image‐analysis.html

**Figure 6:**
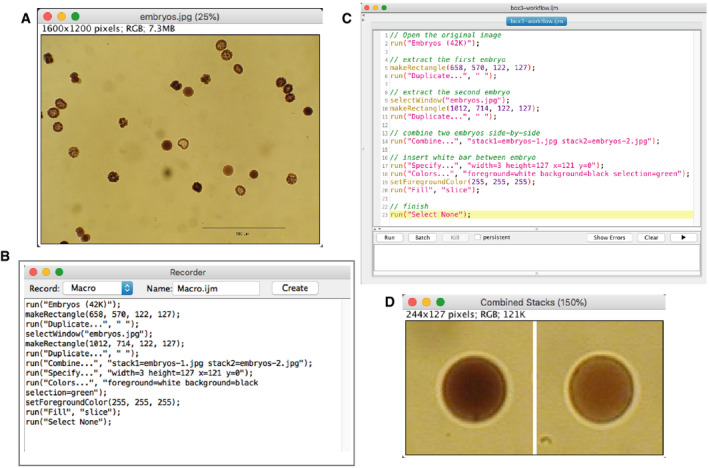
Recording figure creation Proper reporting of image data handling/analysis is facilitated by recording the process of image handling. As an example, we explain a case of creating a figure using the “Command Recorder” function in ImageJ (version 1.51o). (A) The original image. We select two embryos, extract them and create a figure with two panels. (B) The result of macro recording. All these lines were automatically generated during the manual handling of the image. (C) Comments (shown in green) were added manually after recording to clarify what is achieved by each step. Others can easily understand the aim of different parts of the macro by these comments. (D) The figure. The macro shown in (B) can be used to reproduce exactly the same figure from the original image. Macro programming in ImageJ is explained in the chapter “ImageJ Macro Programming” in “Bioimage Data Analysis”, 2016, Wiley‐VCH. The macro code shown here is available at: https://github.com/miura/reproducible_bioimage_analysis.

**Figure 7:**
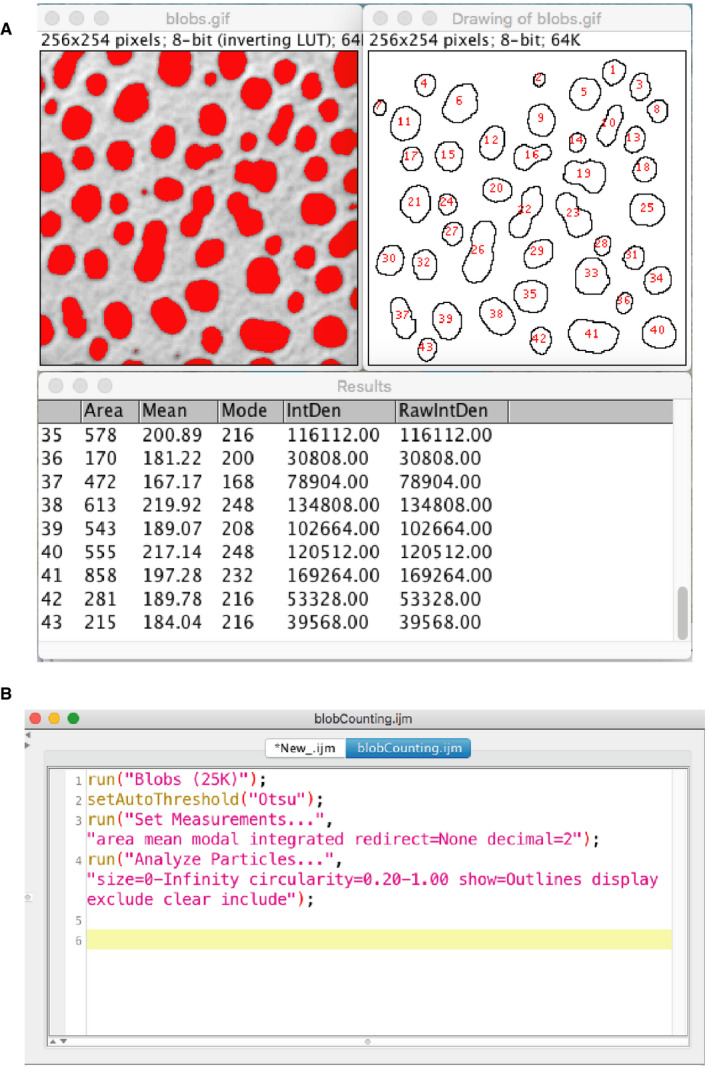
Recording bioimage analysis workflow We show here the recording of a simple analysis workflow as an ImageJ macro. The workflow counts blobs in the sample image “blobs.gif” loaded from the ImageJ menu. We segment blobs with the auto‐threshold function, and then apply particle analysis in order to count the number of blobs and measure their area. Auto‐thresholding of blobs (A, top‐left), segmented blobs (A, top‐right), and the results of particle analysis (a, bottom, the results table). These steps were taken using the GUI of ImageJ, while the command recorder is turned on. (B) The result of command recording of the analysis workflow. The code is slightly cleaned‐up, removing unnecessary steps, and then verified for the reproducibility of results. The code is available in the GitHub repository https://github.com/miura/reproducible_bioimage_analysis.

We therefore recommend software packages that automatically record user actions as text‐based macros or scripts. For example, ImageJ has a utility called a “Command Recorder” that does such recording of GUI actions as lines of text commands. The generated text file, listing commands in a sequential order, is called a “macro” (see Figs 6 and 7). For recording figure creation, as an example, two small regions of the original image (Fig 6A) can be cropped manually (Fig 6D), and during this process, all those manual handlings can be recorded (Fig 6B) and a script can be created based on that recording (Fig 6C). The recording of analysis workflow (Fig 7B), though it can become more complex than figure creation, can also be recorded to generate a macro (Fig 7B). After recording data handling or the analysis workflow as a macro file, users can re‐run the macro to check if the results can be reproduced. If this is ensured, the macro can be used by others for reviewing. Some types of software packages are designed to be used from the command line, such as MATLAB, Python, and R. In this case, the command history can easily be converted to scripts and saved, so these packages by default provide the means for preparing reproducible workflows. Box 2 gives examples of software packages that support the creation of reproducible image analysis workflows.

#### Code as documentation

One important and generally accepted aim of computer programming, or scripting, is to automate repetitive operations in order to minimize the manual workload. However, when it comes to reproducibility, the purpose of programming is different : Here, the goal is to document the workflow precisely and to ensure that the same result can be reproduced by others.

Therefore, the programming of macros or scripts is essential *even if the code is used only once*. We suggest keeping the workflow file as simple as possible, preferably as plain text files. It is also possible to provide a so‐called “container” that fully clones the execution environment, e.g., operating system, installed software and libraries, but we do not explicitly include it as a required component of the workflow package since this technology is emerging and its deployment is still not trivial (Box 3: Shared‐infrastructures for the reproducible analysis). Some web‐based services exist that allow researchers to upload workflow codes and run them on the server‐side. One possible usage of such a service is to enable others to reproduce an image analysis (See also Box 3).

**Box 3. Shared‐infrastructures for the reproducible analysis**

A typical source of problems in reproducing image analysis workflows is the diversity in computational infrastructure. For example, a workflow written in the ImageJ macro language may return errors when an older ImageJ version, or a different Java version, is used. Differences in operating systems may also cause trouble in executing the workflow code. Here, we note several ways to avoid such problems by sharing the infrastructure itself.

1. Use Containers: A software tool called Docker is available now that can be used to create a snapshot of the computational infrastructure originally used for the workflow. That snapshot can then be shared with others to allow them to run the workflow with exactly the same infrastructure used for its implementation. A Docker snapshot preserves the exact version of the software components originally used and can also bundle the text file‐based workflow; therefore, it is an ideal format for delivering reproducible workflows. Since the Docker snapshot can be converted to a readable text file called a Docker file, this also provides good documentation that helps reproducing workflows even if the Docker package itself is not used. Docker is still not generally used in the life science community (it is Unix based with limited Windows support), and we are still evaluating whether to include its use in recommendations for the general publication of reproducible workflows.
2. Use Web Services: Several online services are appearing that allow the public sharing and the execution of workflow on the server‐side, equipped with a web interface. For example, Code Ocean is a commercial service recommended by some publishers (e.g., Nature and EMBO Press). Though the number of use cases is still low, it does solve the problem of infrastructure differences. Apeer is another service recently available promoted by microscope company Zeiss. NEUBIAS has started to provide a web‐service for benchmarking bioimage analysis workflow called BIAFLOW, and this service can also be used as a place to share workflows for others to test (Rubens *et al*, 2020). Though these services are quite useful as there is no problem to occur in terms of the infrastructure, we still cannot be sure how long these services will be kept in the future, sustainable enough for scientific publications.
3. Use Notebooks: Python has a notebook tool called Jupyter, which allows the publishing of workflow codes as server‐side executable documentation. As the notebook can be executed on public servers, and as this is available in popular social code repositories such as GitHub, it is increasingly getting popular to publish workflows in those repositories avoiding the infrastructure problem. A similar notebook tool is also available in R off‐the‐shelf by installing RStudio (R Markdown).

### Workflow description: describe how to reproduce results

Scripts or macros are computer code and should be associated with a text describing the outline of the workflow to explain the overall aim and the key components, as well as instructions on how to run the code (Box 4: Workflow Description). A flowchart, or meta‐code, can be a helpful solution as a visual guide for this outline (Box 4, item 1). If the software is publicly available, it is recommended to place a download link and add notes regarding the installation procedure (Box 4, item 2). Note version numbers of the software tools. When certain components of the workflow require some run‐time input of functional parameters, those values should ideally be included in the description (e.g., as a table) to ensure that others can derive the same results and also to emphasize key parameters (Box 4, item 3, 4, & 5).

**Box 4. Workflow description**

We recommend the following items to be present in the workflow description. Note that for items 1 to 4, the container service (e.g., Docker; See Box 3) allows packaging all the materials together.

1. Outline of the workflow: Bioimage analysis workflows are a set of selected component algorithms that are sequentially applied to the source image (for more details, see (Miura & Tosi, 2018)). The outline describing how these components are assembled will be valuable for others to have a structured understanding of the design of the workflow. A flowchart, such as shown below, will also be helpful for this understanding. Some software, such as ICY, allows the direct visualization of workflow (See for example https://bit.ly/icy‐spot‐detection)
2. Access to the dependent software: The software required for running the workflow must be reported (name, version number and location; a URL is sufficient).
3. Instruction for reproducing the workflow: How to run the code to regenerate the submitted workflow should be explained step‐by‐step.
4. Tables of parameter values used. Preferable in plain text as this is a robust file format. XML or JSON files fall in this category.
5. Details about the original data: biological name of the sample, imaging details (microscope settings), type of the protein label if such markers are used, and space‐time resolution. If the dataset is not a complete dataset, for example because it is too large, this should be noted alongside a description of the complete dataset. Include a download link.

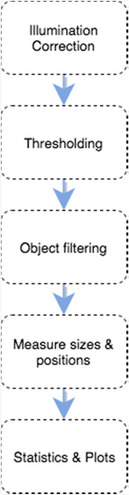

### Original image data: be ready for publication

Collect all image data, which were used for the image analysis workflow, structure them into folders so that the reader can easily associate a specific workflow with the data that they are analyzing. Use a compression tool or data container to package them all in a single file, e.g., ZIP or HDF5. For large datasets that exceed several gigabytes, we suggest preparing a minimally sized *sample* dataset to allow the reviewers and others to test the workflow and to be convinced of the ability to obtain similar results. Both the sample data and the complete data set should be source (raw) data in the format it had when acquired, i.e., before any information reducing processing. For microscopy data, this could mean TIFF files, or a proprietary file format (ND2, LIF, OIF, LSM, CZI, etc). With sample data, the results do not necessarily have to be exactly the same as the complete dataset but it should output similar results that agree well with the conclusion of the full analysis (see Box 1, “Reproducibility”).

In a recent survey, more than 90% of biology PIs report that publishing data is important to them, yet more than half noted that their needs are not met in terms of training and, to a lesser degree, infrastructure (Barone *et al*, 2017). More than half of the respondents work with images, topped only by sequence data. In earlier times, data‐inspired papers without actual data were commonplace. Consider the report on the discovery of the diffusion process by Robert Brown. In his careful study and beautiful exposition of “active molecules” from 1828, the paper contain no data, though it is clearly a data‐driven study. At that time, results were typically disseminated by *reading* one’s own paper to a scientific society; the sharing of raw data was not usual, possibly because of the absence of means to do so. Publishing source data is in fact a largely modern phenomenon; thanks to the development of open‐access software and data‐storage tools.

Here, we might need to clarify that the goal of data publishing in the context of enabling the reproducibility of data handling is slightly different from the data archiving requested by institutions. Funders of scientific research projects are increasingly aware of data archiving, not keeping them only for the verification of results in the future but also as data are seen as results of their investment. Institutional data archiving mandates for the retention of acquired data for a certain period of time has become standard regulation in many countries. Furthermore, the archiving of image data and offering them to the public is now expected to produce a richer outcome based on reuse, re‐analysis, data‐mining, and referencing (Ellenberg *et al*, 2018). On the other hand, for ensuring the reproducibility of data handling and methods, which is the core interest of this article, the scope of data publishing is focused more on sharing the method in a published article with others (Miyakawa, 2020). Practically, the data that need to be published and associated with the workflow can be much smaller in amount than that for the institutional data archiving or with less efforts in preparation compared to the publication aiming for the added‐value database.

### Upload to public data servers

When your workflow package (workflow code, description, and original image data) is ready to be submitted, there are two different forms of submissions for conventional publications: either as supplementary material or an appendix to a paper or, as a stand‐alone method paper, e.g., F1000R NEUBIAS Gateway^3^, or as a preprint in Biorxiv^4^. In all cases, the current format of submission is centered on text material, while other non‐text resources are provided in most cases as download links. We, however, insist that the workflow package should be shared in a publicly accessible location. There is quite some flexibility in choosing the location for publishing these materials online, e.g., at Zenodo^5^, SourceForge^6^, or GitHub^7^ (see Box 5: Data Servers to Share Workflows and Image Data). Links to a university ftp server or dedicated websites containing lists of files are generally discouraged due to their ambiguous persistence. Image databases, such as Image Data Resource (IDR)^8^ and BioStudies (Lemberger, 2015; Sarkans *et al*, 2018)^9^, are excellent places to upload the image dataset when the file size is large and the dataset is intended for reuse by others. In this case, a link to image data can be indicated in the workflow description instead of including the data set in the workflow package. A drawback is that data publishing in these added‐value image databases might be demanding in terms of preparation and peer reviewing, especially when the purpose is limited to provide a specific image data for evaluating a specific workflow.

**Box 5. Data servers to share workflows and image data**

● Zenodo (with Github): Allows free upload of up to 50Gb per dataset together with the workflow description. Code on Github can be linked. It automatically assigns digital object identifiers (DOIs) to data. Versioning is possible.
● GitHub, Bitbucket, Gitlab: For publishing version‐controlled code and descriptions. Space is limited so the upload of image data needs careful consideration.
● Figshare: A commercial, but free, service for sharing figures, datasets, images, and videos.
● The journal: limited space and infrastructure. Also suffers from each journal having its own implementation. Better to find an independent solution.
● RCSB Protein Data Bank and other topic‐specific repositories: if your field has an established repository for sharing data and code use it.
● The Image Data Resource (IDR). Free and public. This database is only for image data with “added‐values” for reuse and re‐analysis. It can take three weeks until the data becomes public. We recommend this repository for datasets exceeding 50Gb and that are meant for further information mining.
● BioImage Archive: A new public database for life science image data. IDR will eventually become integrated into this server.

Currently, we recommend using GitHub and Zenodo to upload and publish reproducible workflow packages because the uploaded package will be associated with a persistent Digital Object Identifier (DOI). It allows stable links from the main paper that you will publish. In Box 6, we provided step‐by‐step Instructions for uploading reproducible bioimage analysis workflow to Zenodo and Github.

**Box 6. Step‐by‐step instructions for uploading reproducible bioimage analysis workflow to Zenodo and Github.**

1. Upload the workflow code to a Github repository. If you do not have an account in Github, create your (free) account and start a new repository for uploading the workflow code. Detailed instructions are available in Github
  ○ https://guides.github.com/activities/hello‐world/#repository).
2. Login to Zenodo using your Github account, and navigate to the Github section within ZENODO site.
  ○ https://zenodo.org/account/settings/github/
  ○ The Github repository you created in Step 1 should be already there in the list. Toggle the switch located to the right of the name of your repository (see the screenshot below).
  ○

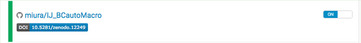

3. Go back to the Github repository page and create a release.
  ○ Follow the instructions in the page below.
  ○ https://help.github.com/articles/creating‐releases/
4. Switch back to the Zenodo page and reload it. It may take a while, but a DOI is now added to your repository. Note the DOI link.
5. Edit the workflow description and add the DOI link to your workflow code acquired in Step 4.
6. In Zenodo, click “upload” at the top bar of the page. Drag and drop the workflow description and the zipped original image data archive. Uploading starts.
7. The DOI for this repository will be the identifier of your reproducible bioimage analysis workflow, that can now be cited directly.

## Recommendations for Editors

A recent paper examined the level of reproducibility of analysis results published in the journal Science (Stodden *et al*, 2018) and found, empirically, that the analysis performed in 26% of about two hundred randomly selected papers could be reproduced. We are not aware of any systematic study of the frequency of reporting image analysis methods, among papers that rely on such analysis. Arguably, the rate of reporting image analysis methods will be less than or equal to the rate of reporting image acquisition methods, since acquisition comes earlier in the experimental pipeline and tends to be easier to report. For image acquisition, we know that the rate of reporting is currently low, on average about 17% and as low as 3% at some journals (Marqués *et al*, 2020).

Thus, instead of trying to design a perfect "image data handling and analysis" rule‐set as a part of journal policy, evaluating this aspect of the submitted papers solely for the reproducibility of the analysis and its results will be more effective in avoiding accidental mistakes and in preventing intentional misconduct. To promote the submission of reproducible bioimage analysis workflows, we recommend that journal editors ask researchers to evaluate *themselves* the reproducibility of their image handling and analysis in the submitted paper. A table that may help during self‐evaluating of reproducibility is shown in Table 1. The traditional approach has been “Barely” reproducible description, data, and code. At the other end of the spectrum, we have “Fully” reproducible image data handling, now available to virtually anyone, thanks to the availability of open‐access data‐storage platforms. A group of journal editors are making an effort in a similar direction to set the minimal standards for materials including data and code, design, analysis and reporting (preprint: Chambers *et al*, 2019). We hope that our list may become a good reference for setting such a standard.

**Table 1:** Self‐evaluation of image analysis reproducibility level. Three levels of reproducibility that authors are encouraged to use to classify their manuscript, at the time of submission, for each element of the performed image analysis workflow. See also Box 2 for requirements on the software used

Reproducibility	Description	Software	Data	Workflow Code
Fully (The scientific approach)	Complete instructions for running the workflow code is provided using original image data, with specified tools and their version. Information about the IT hardware used is also provided if relevant. Instructions are given in the manuscript, supplementary information, or a separate publicly accessible resource.	All software used is freely available and open source.	All raw data used that were analyzed to reach the conclusions reported in the paper and its supplementary information are available online.	Image analysis and figure creation workflow codes are available directly online in the version used for the manuscript to allow reproduction of all results and figures.
Largely (Well intended, could do better)	A detailed description of image analysis steps taken is provided, in the manuscript or as supplementary material. An interested reader can probably reproduce the analysis.	Software may be commercial or closed‐source or available upon request.	Some or all of the data is available upon request along the data‐sharing guidelines of the journal.	Image analysis and figure creation workflow codes are available to others in some way to allow reproduction of all or some parts of results and figures.
Barely (“Trust me, I’m a scientist” approach)	Only states what software was used and/or a rough outline of steps taken. More information might be provided by authors upon well‐motivated request.	Software is not publicly available.	No data provided beyond what appears as tables and figures in the manuscript.	Does not exist, or might be obtainable by directly contacting authors.

We do see positive changes in the process of academic publication that may give rise to more awareness in the reproducibility of methods. The preprint posting of a manuscript might help obtain feedback ahead of publication to improve reporting standards. These trends could evolve into a more dynamic research paper, with continuous commenting / reviewing of the online paper, and further revision cycles based on the feedback. We can see this as a revision‐tracked academic paper akin to version‐controlled software releases.

The complexity of the methods used in each paper is increasing. More advanced methods in bioinformatics, genomics, proteomics, statistics, and imaging are used in combination in a single paper, rendering them harder to be assessed by the typical number of two to three referees. For example, deficiencies in the statistical methods used for clinical research were reported already twenty years ago, and inclusion of dedicated statistics experts in the reviewing process was proposed (Goodman *et al*, 1998). A more open publication process with commenting, feedback, and revisions may facilitate the involvement of more experts, specializing in each of the relevant technological modalities used in the submitted papers. For example, members from the Network of European Bioimage Analysts (NEUBIAS neubias.org) could provide expert feedback on image analysis methods. Papers lacking reproducible methods could be flagged by experts, and the revised version should then gain reproducibility.

## Conclusion

Misconduct, in the handling and analysis of image data, comes in two distinct flavors: The first, more accidental, is caused by lack of knowledge about image analysis, when utilizing image data in scientific research. The other, intentional misconduct, is caused by the deliberate fabrication and misrepresentation of data, for the sake of a boost in publication impact, at the cost of scientific integrity and potentially misdirecting the research of peers. In both cases, adoption of best practices, in publishing reproducible image handling and analysis, will increase both the transparency and impact of the research. Employing detailed do‐and‐don’t rules can only have limited positive effects, especially compared to the more robust approach of ensuring reproducibility. Therefore, our approach here has been to discuss the underlying principles and general operational approaches that we believe to be central to reproducibility in image analysis. We hope that this commentary encourages the use of the many powerful image analysis methods available in life science research, with only one simple rule: *The image analysis workflow should be fully documented and reproducible*.

## Conflict of interest

The authors declare that they have no conflict of interest.

## Supporting information



Source Data for Boxes

Original_Images

## Notes

^1^ e.g. Image integrity & Standards, Nature Methods, accessed on Mar. 12, 2018 https://www.nature.com/authors/policies/image.html

^2^
https://forum.image.sc/

^3^
https://f1000research.com/NEUBIAS

^4^
https://www.biorxiv.org

^5^
https://zenodo.org

^6^
https://sourceforge.net

^7^
https://github.com

^8^
http://idr.openmicroscopy.org, only for image data

^9^
https://www.ebi.ac.uk/biostudies/
