# Supplementary figures and images for: Reproducible image handling and analysis
Source: EMBO J. 2021 Jan 22;40(3):e105889. doi: 10.15252/embj.2020105889 (PMC7849301; doi:10.15252/embj.2020105889)

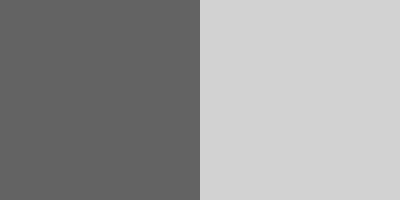

Supplement: Supplementary file 1 — Source Data for Boxes [file EMBJ-40-e105889-s001.zip › EMBOJ-2020-105889_Original_Images/Box4/EMBOJ-2020-105889_box-4_enhanced.png]

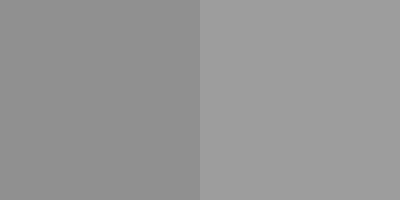

Supplement: Supplementary file 1 — Source Data for Boxes [file EMBJ-40-e105889-s001.zip › EMBOJ-2020-105889_Original_Images/Box4/EMBOJ-2020-105889_box-4_original.png]

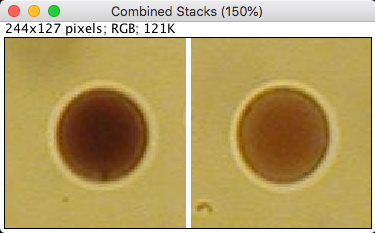

Supplement: Supplementary file 1 — Source Data for Boxes [file EMBJ-40-e105889-s001.zip › EMBOJ-2020-105889_Original_Images/Box6.1/EMBOJ-2020-105889_box6-1_figure.png]

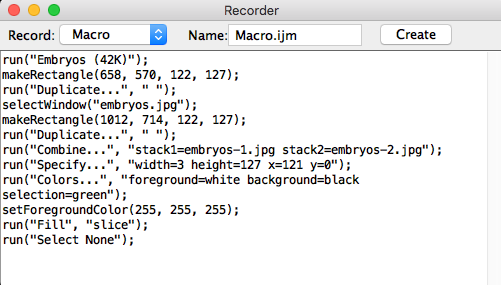

Supplement: Supplementary file 1 — Source Data for Boxes [file EMBJ-40-e105889-s001.zip › EMBOJ-2020-105889_Original_Images/Box6.1/EMBOJ-2020-105889_box6-1_recorder_results.png]

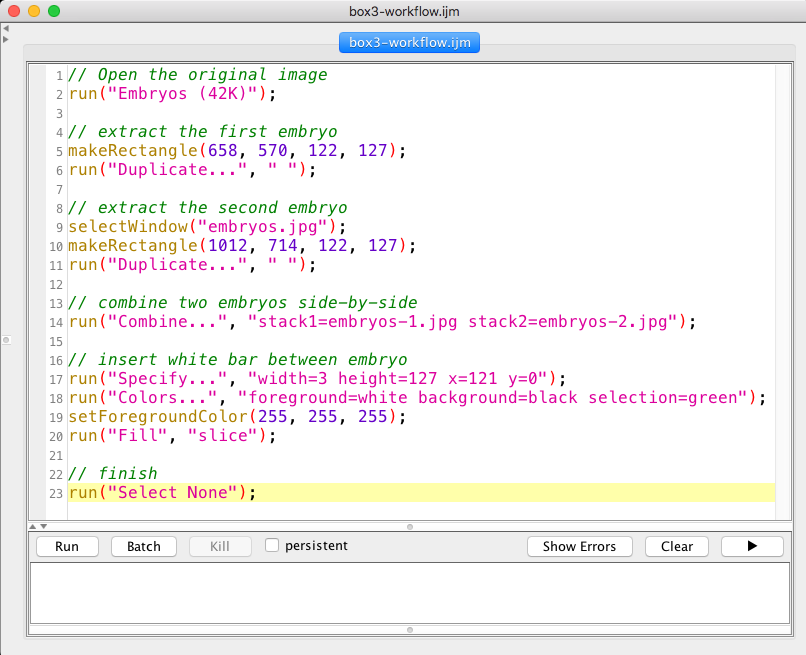

Supplement: Supplementary file 1 — Source Data for Boxes [file EMBJ-40-e105889-s001.zip › EMBOJ-2020-105889_Original_Images/Box6.1/EMBOJ-2020-105889_box6-1_workflowEditor.png]

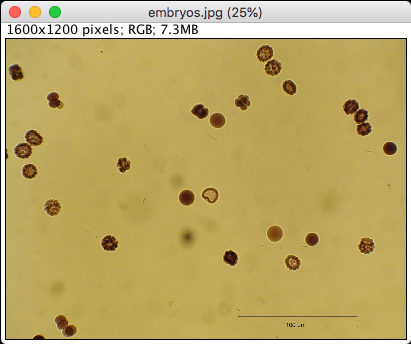

Supplement: Supplementary file 1 — Source Data for Boxes [file EMBJ-40-e105889-s001.zip › EMBOJ-2020-105889_Original_Images/Box6.1/EMBOJ-2020-105889_box6-1_embryos.png]

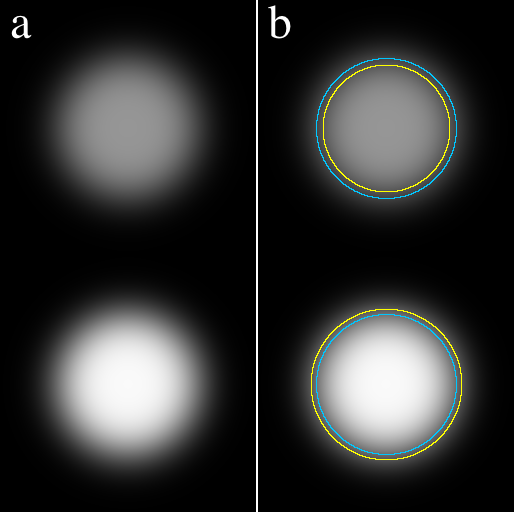

Supplement: Supplementary file 1 — Source Data for Boxes [file EMBJ-40-e105889-s001.zip › EMBOJ-2020-105889_Original_Images/Box3.2/EMBOJ-2020-105889_Box3-2_circles_figure.png]

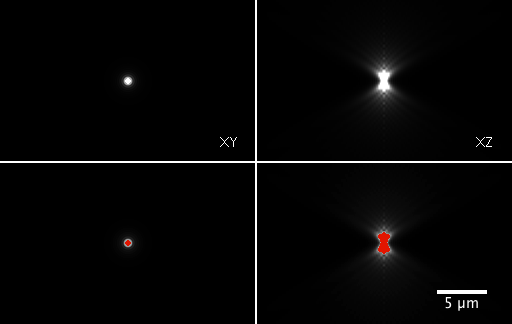

Supplement: Supplementary file 1 — Source Data for Boxes [file EMBJ-40-e105889-s001.zip › EMBOJ-2020-105889_Original_Images/Box3.4/EMBOJ-2020-105889_box3-4figure.png]

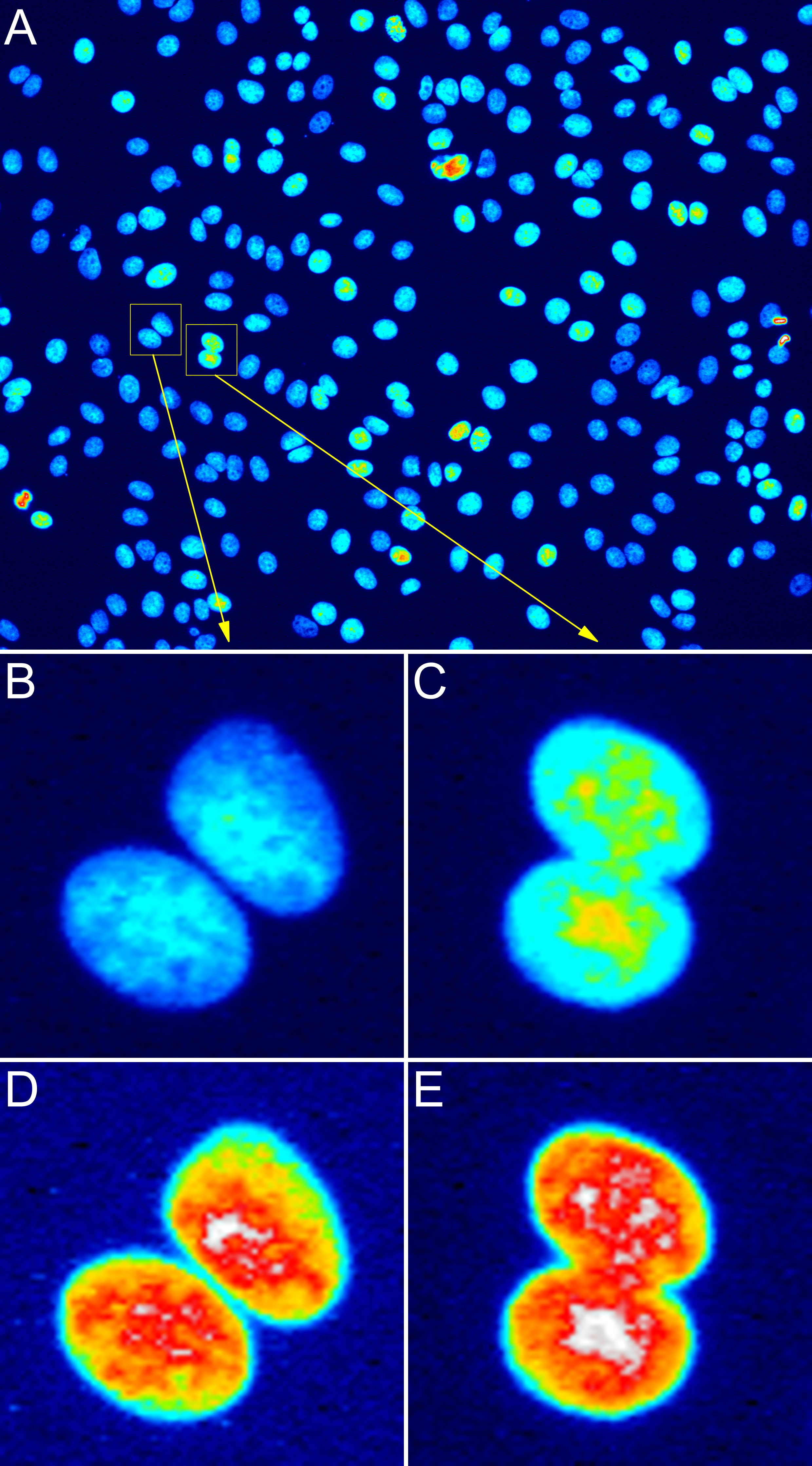

Supplement: Supplementary file 1 — Source Data for Boxes [file EMBJ-40-e105889-s001.zip › EMBOJ-2020-105889_Original_Images/Box3.3/EMBOJ-2020-105889_Box3-3_scientifig_v1.png]

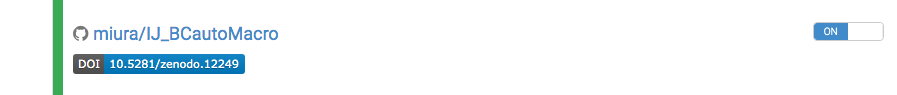

Supplement: Supplementary file 1 — Source Data for Boxes [file EMBJ-40-e105889-s001.zip › EMBOJ-2020-105889_Original_Images/Box9/EMBOJ-2020-105889_box9_ZENODO_example.png]

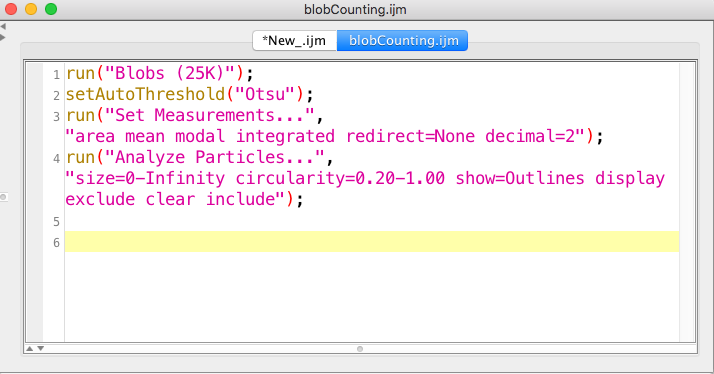

Supplement: Supplementary file 1 — Source Data for Boxes [file EMBJ-40-e105889-s001.zip › EMBOJ-2020-105889_Original_Images/Box6.2/EMBOJ-2020-105889_box6-2_IJcodes_workflow.png]

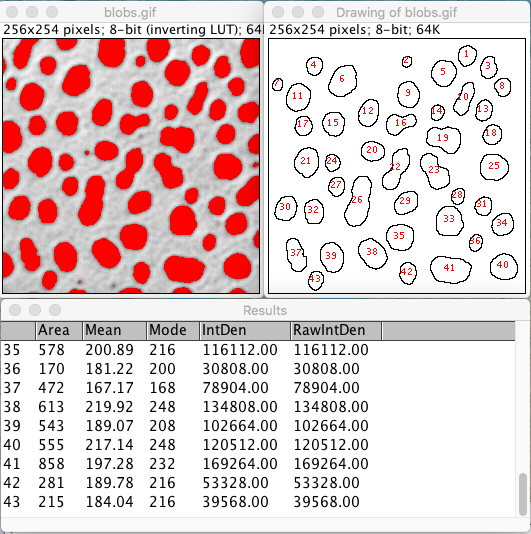

Supplement: Supplementary file 1 — Source Data for Boxes [file EMBJ-40-e105889-s001.zip › EMBOJ-2020-105889_Original_Images/Box6.2/EMBOJ-2020-105889_box6-2_outputs.png]

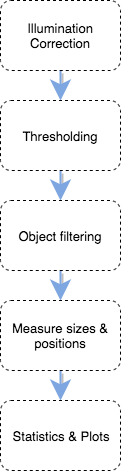

Supplement: Supplementary file 1 — Source Data for Boxes [file EMBJ-40-e105889-s001.zip › EMBOJ-2020-105889_Original_Images/Box8/EMBOJ-2020-105889_Box8_flowchartEG.png]

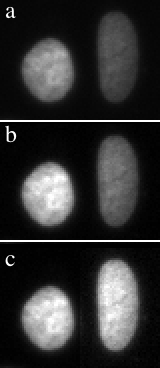

Supplement: Supplementary file 1 — Source Data for Boxes [file EMBJ-40-e105889-s001.zip › EMBOJ-2020-105889_Original_Images/Box3.1/EMBOJ-2020-105889_Box3-1_EC_twoways.png]

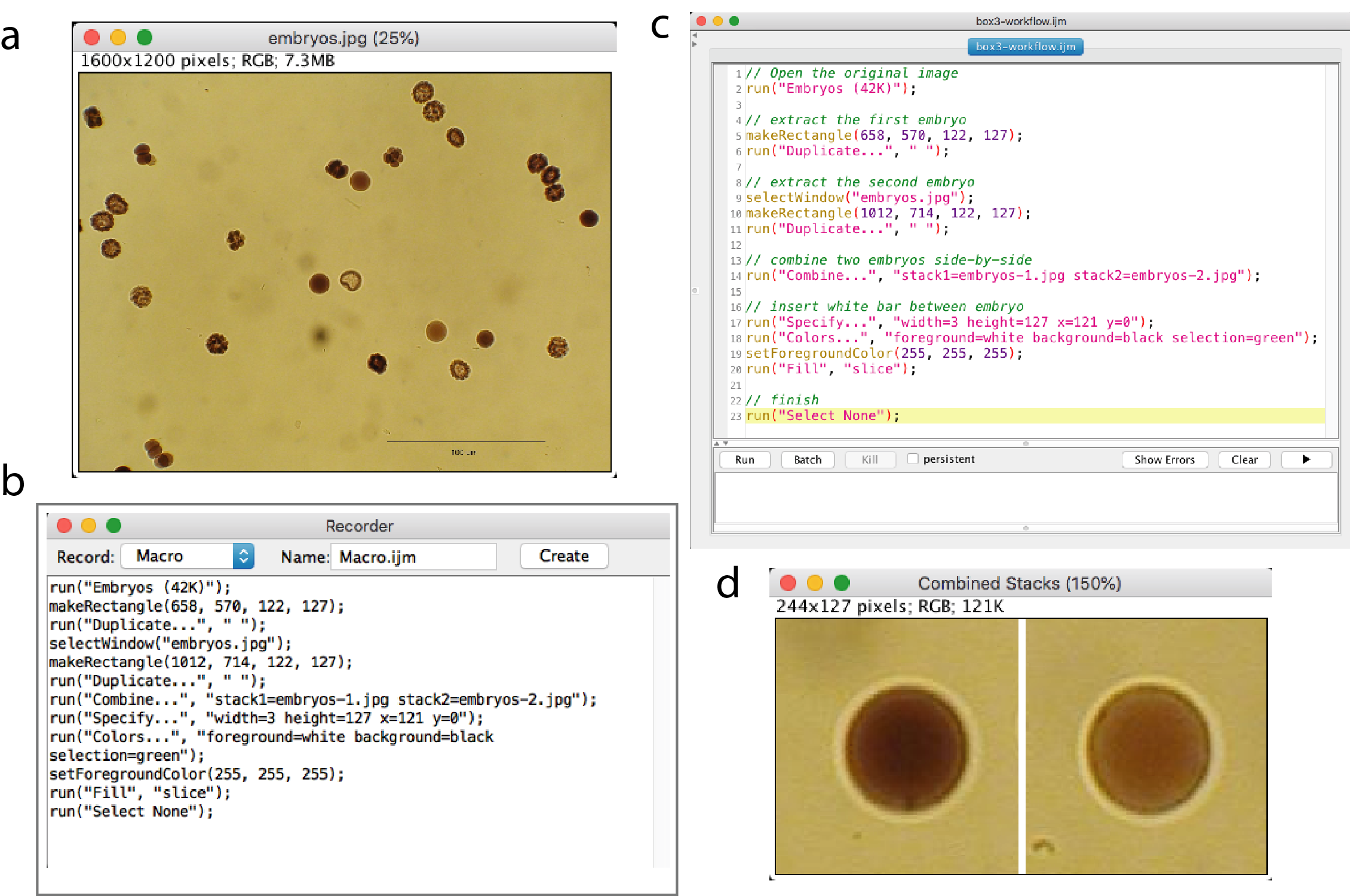

Supplement: Supplementary file 2 — Original_Images [file EMBJ-40-e105889-s002.zip › EMBOJ-2020-105889_Original_Images/Fig06/EMBOJ-2020-105889_Fig06figuremacro.png]

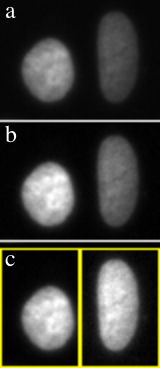

Supplement: Supplementary file 2 — Original_Images [file EMBJ-40-e105889-s002.zip › EMBOJ-2020-105889_Original_Images/Fig01/EMBOJ-2020-105889_Fig01_EC_twoways.png]

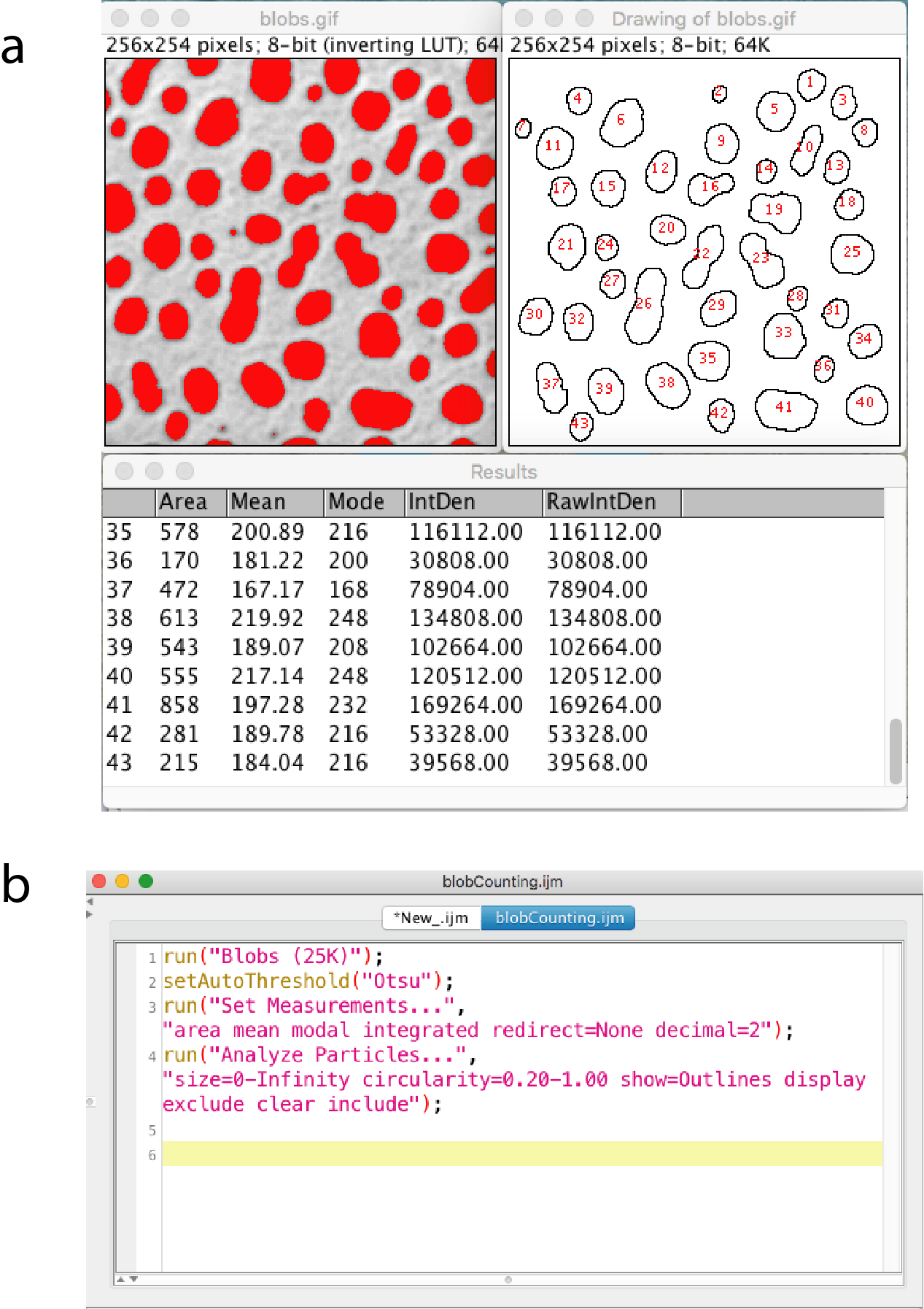

Supplement: Supplementary file 2 — Original_Images [file EMBJ-40-e105889-s002.zip › EMBOJ-2020-105889_Original_Images/Fig07/EMBOJ-2020-105889_Fig07workflowImages.png]
